# Accumulating the key proteomic signatures associated with delirium: Evidence from systematic review

**DOI:** 10.1371/journal.pone.0309827

**Published:** 2024-12-19

**Authors:** Md Parvez Mosharaf, Khorshed Alam, Jeff Gow, Rashidul Alam Mahumud

**Affiliations:** 1 School of Business, Faculty of Business, Education, Law and Arts, University of Southern Queensland, Toowoomba, Queensland, Australia; 2 Bioinformatics Lab, Department of Statistics, University of Rajshahi, Rajshahi, Bangladesh; 3 School of Accounting, Economics and Finance, University of KwaZulu-Natal, Durban, South Africa; 4 NHMRC Clinical Trials Centre, Faculty of Medicine and Health, The University of Sydney, Camperdown, New South Wales, Australia; Fukuoka University, JAPAN

## Abstract

Delirium is a severe neuropsychiatric illness that occurs frequently in intensive care and postoperative units which results in prolonged hospital stays and increases patient’s mortality and morbidity rates. This review focused on accumulating the common key proteomic signatures significantly associated with delirium. We carried out a systematic literature review of studies on delirium proteomic biomarkers published between 1^st^ January 2000 and 31^st^ December 2023 from the following electronic bibliographic databases including PubMed, Scopus, and EBSCOhost (CINAHL, Medline). A total of 1746 studies were identified and reviewed, and 78 studies were included in our review. The PRISMA guidelines, the PEO framework, and JBI quality assessment method were followed in this review to maintain the inclusion and exclusion criteria and risk of bias assessment. Most of the included studies were of the cohort (68%) and case-control (23%) design. We have accumulated a total of 313 proteins or gene encoded proteins of which 189 were unique. Among the unique proteins, we focused on the top 13 most investigated proteins (IL-6, CRP, IL-8, S100B, IL-10, TNF-a, IL-1b, Cortisol, MCP-1, GFAP, IGF-1, IL-1ra, and NFL) that are significantly associated with delirium. Most of these are cytokines and inflammatory proteins indicating a strong interconnection with delirium. There was remarkable inconsistency among the studies in reporting the specific potential proteomic biomarker. No single proteomic biomarker can be solely used to diagnose and predict delirium. The current review provides a rationale for further molecular investigation of delirium-related proteomic biomarkers. Also, it’s recommended to conduct further in-depth molecular research to decipher drug target biomolecules for potential prognostic, diagnostic, and therapeutic development against delirium.

## Introduction

Delirium is regarded as a multifactorial medical condition, and its underlying pathologies might be caused by trauma, stress, or inflammation. Delirium is a severe but treatable medical disorder that has been known for more than 2500 years. More than 30 terms have been used to describe it, including disturbance in attention and consciousness which tends to oscillate for a short term [1, 2]. Delirium is often poorly diagnosed and remained largely unrecognized among hospitalized patients, particularly in intensive care units (ICU) [3–5]. Delirium in the elderly is becoming more common, affecting up to 50% of adult hospitalized patients [6–8]. Delirium has a significant impact on a patient’s recovery and increases complications in hospital settings, which extend hospital stays, raise overall costs, and increases mortality [9]. The three main hypotheses for delirium development and its progression include the alteration of neurotransmitter systems, the activity of inflammatory cytokines leading to permeabilization of the blood-brain barrier, and disruption of the hypothalamic-pituitary axis in response to severe trauma [10].

Yet, delirious patients in ICU/hospital settings may benefit from additional biological, molecular, and pathophysiological insights provided by molecular biomarkers associated with delirium incidence [11]. Genetic biomarkers are mainly classified into three basic groups: risk markers, disease markers, and end-products. The related biomarkers of delirium have been identified by several systematic reviews, and these biomarkers include distinct cerebrospinal fluid, amino acids, proteins, genes, regulatory molecules, genetic variation (i.e., SNIP), and other molecules as well [12–14]. Despite some discrepancies in the results, the identified biomarkers thus so far are internally linked by known functional interactions and molecular pathways [12]. Over the past few decades, the complexity of the molecular network-based biological functions and pathomechanisms influencing delirium development and its severity have been identified. There remains a knowledge gap about genetic factors, their regulatory elements, functional and molecular pathways, and the pathomechanisms of delirium origin and progression.

It has been observed that the pathophysiology of delirium and its complications in medical settings remain unknown based on the body of existing literature [15]. The molecular investigation is one of the effective and modern techniques that may assist with diagnosis, evaluation, and treatment while also shedding light on its mysterious pathogenesis [11, 16, 17]. In this aspect, the proteomic biomarkers efficiently indicate the severity, risk, onset, and recovery of the disease and disease motion. They can be treated as a potential therapeutic target for drug development [18, 19]. Even though delirium has been linked to certain biomarkers, research has revealed conflicting results, leaving no clear biomarkers for delirium. Yet, only a few studies have been conducted to accumulate the molecular proteomic biomarkers of delirium, indicating a lack of knowledge about this critical medical condition.

Therefore, this study focused on accumulating and identifying the key common proteomic signatures associated with delirium that have been studied so far. The review also justified the proteomic functional diversity of the common proteins associated with delirium. In addition, we have provided a comprehensive summary of the current state of knowledge on the proteomic signatures of delirium, which may form the basis of future in-depth molecular research and ultimately help with the development of more effective and potent drugs for delirium treatment.

## Materials and methods

### Systematic review

We conducted a systematic analysis of the literature to identify research on delirium-associated proteomic biomarkers. The entire procedure was guided by the Preferred Reporting Items for Systematic Reviews and Meta-Analyses (PRISMA) standards, [20] and associated PRISMA flowchart. The search strategy, inclusion and exclusion criteria utilized the PEO framework described below [21]:

**P**opulation: The study population solely included confirmed cases of delirium in humans. **E**xposure: The delirium-associated proteins/gene-encoded proteins which are the biomarker proteins that are significantly associated with delirium. **O**utcome:–this was delirium, and the identification of significant proteomic biomarkers associated with delirium. Study design—In the systematic review, studies from all kinds of observational and experimental were considered. This review was registered on PROSPERO (registration number: CRD42024566515).

### Search strategy

A comprehensive electronic literature search was conducted on the selected electronic bibliographic databases (PubMed, Scopus, and EBSCOhost (CINAHL, MEDLINE)) using the MeSH terms, keywords, and subject headings. Only studies that were published in journals between 1^st^ January 2000 and 31^st^ December 2023 were considered for screening. The primary keywords were “delirium” and “biomarkers” used along with a combination of other associated keywords including “markers”, ‘genetics”, “genes” and “proteins” to search the studies. Boolean operators “AND”, and “OR” were applied to combine the searching keywords. In addition, this review was complemented by a thorough manual search of related studies. Further, studies were identified through citation searches of included studies and manual searches for professional web sources and key journals in these fields of research. The details of the search sentences used in different databases and their search outcomes are provided in S1 File.

### Eligibility criteria

Eligible studies were included if they were i) based on original research studies focused and reported on genes/proteins showing any statistically significant relationship between delirium and genes/proteins in human cases; ii) delirium was assessed and confirmed using established delirium assessment methods; and iii) were published between January 1, 2000, and December 31, 2023, in English. Otherwise, editorials, letters, perspectives, commentaries, reports, reviews and meta-analysis, study protocols, publications in other languages, and studies with ’insufficient related data’ were excluded.

### Study screening and selection process

The eligibility of studies to be included was determined following a three-stage screening process. The first stage involved screening of studies by title to eliminate duplications. The second stage required reading abstracts to determine their relevance to our study. Finally, the third stage necessitated reading full texts of the retained studies, and those that met the set criteria were kept. After screening the title and abstract, MPM and RAM carried out the full text screening to select the articles. During this process, we have discussed and reached a consensus with the other authors (KA and JG) to resolve any discrepancies.

### Quality assessment

Quality assessment of the 78 included studies was conducted because of the heterogeneity among the study designs of the included studies. In this systematic review, cohort, case-control, cross-sectional, randomized control trials, and longitudinal study designs were found among the included studies. The Joanna Briggs Institute (JBI) [22] provided critical quality assessment tools that have been utilized in this study for quality assessment. The JBI quality appraisal tools are widely used in academic studies to assess the risk of bias (graded as high, moderate, or low) [23–26] where the higher quality scores demonstrate better confidence and vice versa. The JBI appraisal tool was used to evaluate the 53 cohort studies, 18 case-controls, three randomized control trials, two cross-sectional, and two longitudinal studies included in our review. The overall quality appraisal scores are summarized in S2 File.

### Data extraction

The data were extracted from Mendeley libraries by one researcher (MPM) with the direct help and guidance of RAM, who subsequently reviewed the results. Discrepancies in the data were addressed and resolved by consensus, and in cases where the two researchers could not reach a consensus, other researchers (KA and JG) were consulted for adjudication. The studies reported the genes and proteins significantly related to delirium were mainly considered for this qualitative synthesis. At the data extraction stage from the selected articles, we considered the first author, publication year, age, gender, data collection time, method of detecting proteins/genes, country of study, study design, method of delirium assessment, and the reported significant proteins associated with delirium. Any missing information was kept blank or noted by *“NA”* on the data extraction table. The entire procedure was guided and completed by the systematic literature review tool Covidence (https://app.covidence.org).

## Results

### Description of included studies

**Fig 1:**
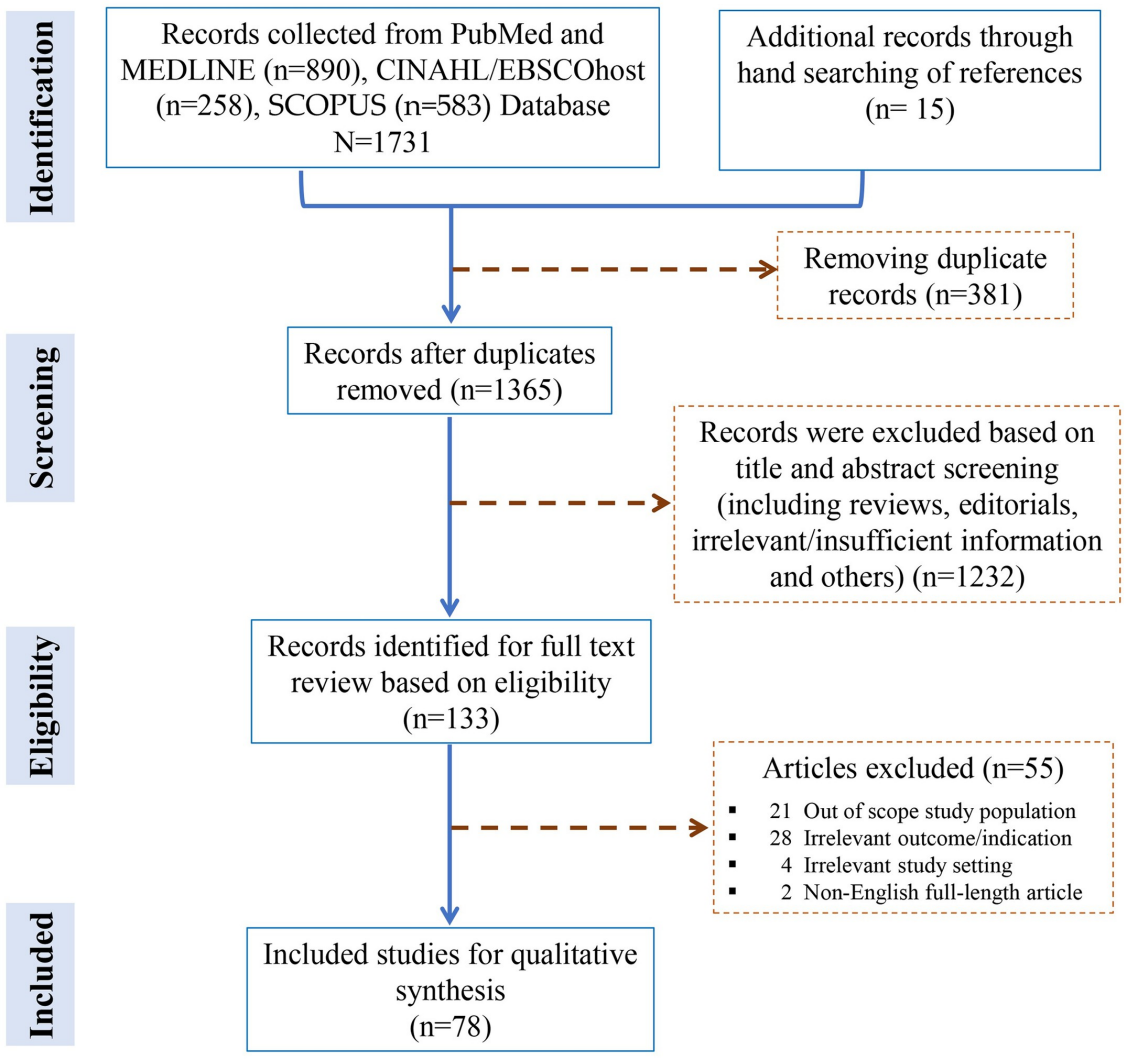
The PRISMA flow diagram of this study.

Our search identified 1746 records, of which 1365 were screened (Fig 1). After removing 381 duplicates, 1365 records were excluded at title and abstract screening stage including for example, review papers, editorials, protocols, irrelevant and insufficient information, and others. Finally, 133 full-text studies were assessed for eligibility, and 78 studies were included in the systematic literature review (Fig 1). Fig 1 demonstrates the PRISMA flowchart and the PRISMA Checklist has been provided in S3 File.

**Fig 2:**
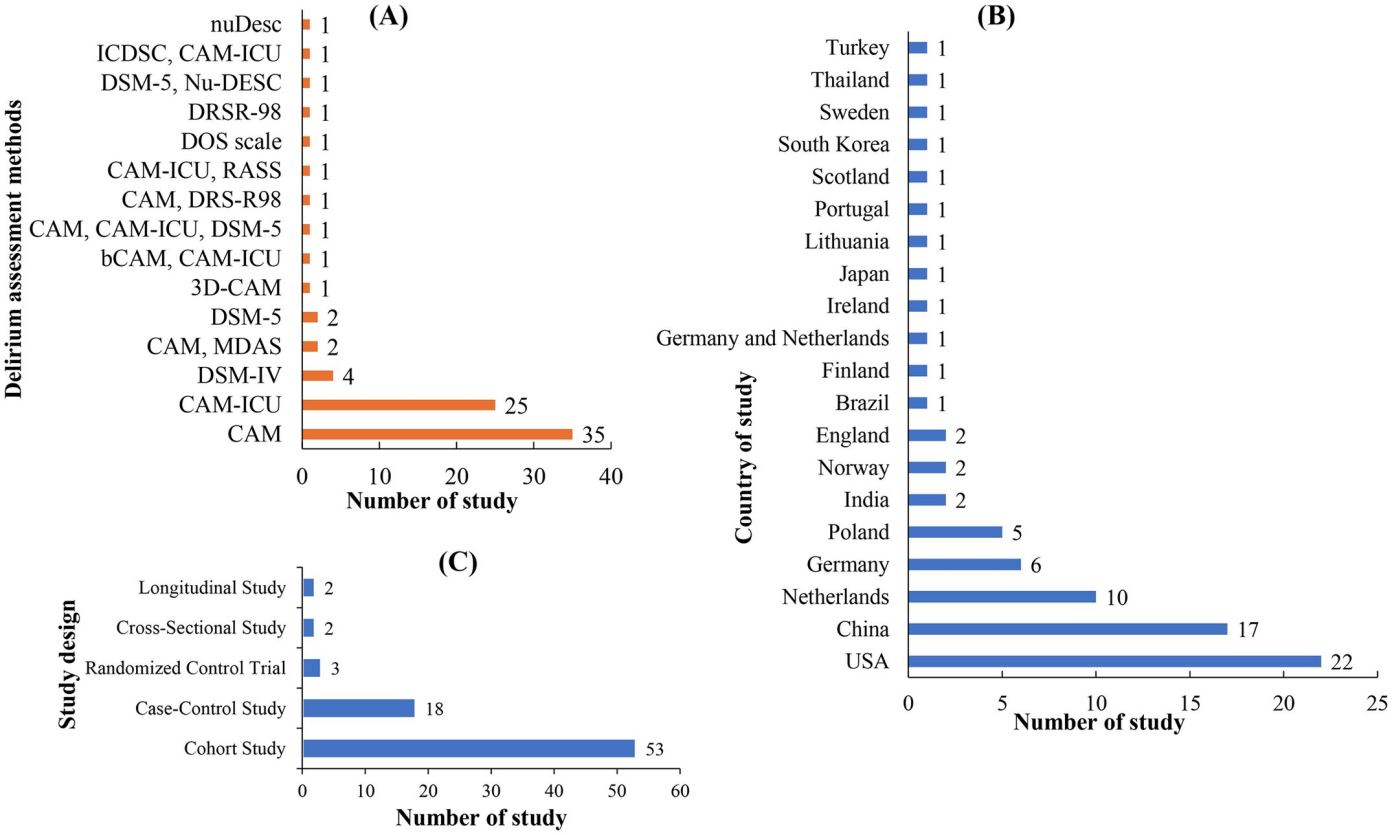
Distribution of the included studies according to (A) the delirium assessment method, (B) study design, and (C) country of study. [Here, (b)(3D)CAM: (Brief) (3 minutes) Confusion Assessment Method; CAM-ICU: Confusion Assessment Method for ICU; DSM-IV and DSM-5: Diagnostic and Statistical Manual of Mental Disorders, 4th and 5th edition; DOS Scale: Delirium Observation Scale, DRSR-98/DRS-R98: Delirium Rating Scale-Revised-98; NuDesc: Nursing Delirium Screening Scale; ICDSC: Intensive Care Delirium Screening Checklist; RASS: Richmond Agitation Sedation Scale; MDAS: Memorial Delirium Assessment Scale].

The included studies differed significantly in terms of patients, research methodology and settings, and the biomarker investigated (Fig 2). In terms of context, participants were from either a medical or surgical environment setting. In the delirium-only trials, authors either did not include patients with comorbidities or did not assess neurocognition to ascertain whether comorbidities were present or not. Studies with additional comorbidities did not consistently account for these variables’ existence. Pre-existing cognitive impairment and Alzheimer’s disease were considered as dementia in this study and hence excluded.

All 78 studies have utilized different well-established delirium assessment methods to identify delirium either in the pre-operative or postoperative stage for critically ill patients. Twelve different delirium assessment methods were used by the included studies. Among them most of the studies used the Confusion Assessment Method (CAM; n = 35 studies) or its imitation for ICU (CAM-ICU; n = 25 studies) for delirium screening (Fig 2A). Other studies utilized the Diagnostic and Statistical Manual of Mental Disorders, 4th edition (DSM-IV; n = 4 studies) and Delirium Observation Scale (DOS), Delirium Rating Scale-Revised-98 (DRSR-98) and Nursing Delirium Screening Scale (NuDesc) were utilized by three single studies individually. A few studies were also utilized multiple assessment method to diagnose delirium (Fig 2A). Among the selected studies, most of them were conducted in the USA (n = 22 studies), followed by China (n = 17 studies), the Netherlands (n = 10 studies), the Germany (n = 6 studies), the Poland (n = 5 studies), the India (n = 2 studies) and the Norway (n = 2 studies), and other 11 different countries (Fig 2B). The included studies have a diversity of study settings including n = 53 (68%) cohort studies, n = 18 (23%) case-control studies, and other study settings as well (Table 1 and Fig 2C). The patient’s demographic and study characteristics, including age, gender, sample size, number of delirious cases, and methods used to identify proteins/genes, have been documented in S1 Table. The screened full-text studies with decision, entire data matrix have been recorded in S2 Table. The authors’ name and publication year, country of the study, type of study, delirium assessment method and the reported proteins/gene encoded proteins have been summarized in Table 1.

**Table 1:** Summary information of the 78 selected studies.

Authors & year	Country	Type of Study	Delirium Assessment Methods	All Associated Proteins/Genes
Adamis et al, 2009 [27]	Ireland	Longitudinal Study	CAM	IGF-1, IL-1ra, IFN-γ
Egberts et al, 2015 [28]	Netherlands	Case-Control Study	DSM-IV	Neopterin, IL-6, IGF-1
Hirsch et al, 2016 [29]	USA	Cohort Study	CAM	IL-5, IL-6, RAGE, IL-8, MCP-1, IL-10, IFN-a, IL-4, IFN-γ, IL-12, TNF-a, MIP-1α, MIP-1β
Kazmierski J et al, 2014 [30]	Poland	Cohort Study	CAM-ICU	IL-2, TNF-a
Miao et al, 2018 [31]	China	Cohort Study	DSM-IV	Neopterin, CRP, IL-6, IGF-1
Ritter et al, 2014 [32]	Brazil	Cohort Study	CAM-ICU	STNFR1, STNFR2, adiponectin, IL-1b
Sun et al, 2016 [33]	China	Cohort Study	CAM	IL-6, CRP, procalcitonin, Cortisol, ABI-40
**Van Munster et al, 2008** [34]	Netherlands	Cohort Study	CAM	IL-6, IL-8
Vasunilashorn SM et al, 2015 [35]	USA	Case-Control Study	CAM	IL-6, IL-2, TNF-a, IL-12, VEGF
**Liu X et al, 2023** [36]	China	Case-Control Study	CAM-ICU	NFL, GFAP
Sun Y et al, 2023 [37]	China	Cohort Study	CAM	CRP
Oren RL et at, 2023 [38]	USA	Cohort Study	3D-CAM	IL-8, LTBR, IL-6, ASGR1
Wu X et al, 2023a [39]	China	Cohort Study	CAM	Aβ40, Aβ42, P-Tau
Ruhnau J et al, 2023 [40]	Germany	Cohort Study	DSM-5, Nu-DESC	sTREM2, Gasdermin D, IL-6, S100B, IL-1B
Westhoff et al, 2013 [41]	Netherlands	Cohort Study	CAM	IL-6, IL-1ra, FLT-31
Heinrich M et al, 2021 [42]	Germany and Netherlands	Cohort Study	CAM	CHRM2, CHRM4
Van Munster et al, 2010 [43]	Netherlands	Cohort Study	CAM	DRD2, DRD3, SLC6A3
Terrelonge M et al, 2022 [44]	USA	Case-Control Study	CAM	FKBP5, KIBRA, KLOTHO, MTNR1B, SIRT1
Yamanashi T et al, 2021 [45]	USA	Cohort Study	CAM-ICU	TNF
Yamanashi T et al, 2021 [46]	USA	Cohort Study	CAM-ICU	TNF-a, IL-1b, IL-6
Steimer M et al, 2021 [47]	Germany	Cohort Study	nuDesc	PER2, HO1
Nekrosius D et al, 2019 [48]	Lithuania	Cohort Study	CAM	COMT
Rhee J et al, 2021 [49]	USA	Randomized Control Trial	CAM	FN1.4, FN1.3, Troponin-1, C5a, IL-1, Cadherin-12, IL-6, PKC-Z, FGF-16, TIMP-1
**Ballweg T et al, 2021** [50]	USA	Cohort Study	CAM-ICU	IL-8, IL-10, MCP-1
Tang C et al, 2020 [51]	China	Randomized Control Trial	CAM	IL-6, TNF-a, IL-10
Vasunilashorn SM et al, 2019 [52]	USA	Case-Control Study	CAM	IL-6, IL-2, CRP, SERPINA3, HPX, ORM1, AZGP1
**Nübel J et al, 2023** [53]	Germany	Cohort Study	CAM-ICU	NSE
Dillon St et al, 2023 [54]	USA	Case-control Study	CAM	ACAN, CFL1, CXCL11, H2AFZ, MUC1, NAMPT, INS, CD97, ICOS, PARK7, FAM107B, CD38, NGF, PPIF, THPO, DCN, MICA, HAPLN1, CTSV, GNLY, CXCL6, CCL2, MMP14, MSN, CHRDL1, PROC, CCL28, CTSD, FSTL1, IGFBP2, PTPN6, PRKCA
Zhang Y et al, 2023 [55]	China	Longitudinal Study	CAM	IL-6, sIL-6R
Liang F et al, 2023 [56]	USA	Cohort Study	CAM, MDAS	Tau-PT181, Tau-PT217
Leung JM et al, 2023 [57]	USA	Case-Control Study	CAM	NFL
**Van Munster BC et al, 2010** [58]	Netherlands	Cohort Study	CAM	Cortisol, IL-6, IL-8, S100B
**Peters van Ton AM et al, 2020** [59]	Netherlands	Case-Control Study	DSM-IV	NBL1, THY1, NrCAM, NCAN, TNKRSF21, DINER, CADM3, RGMA, CTSS, IL-1a, RSPO1, N-Cdase, WFIKKN1, HAGH, CD200R1, IL-5ra, ENRAGE, MCP4, CRTAM, NEP, CST5, BCAN, CASP8, EFNA4, SCF, EZR, CX3CL1, HGF, TGF-B1, SMOC2, CXCL6
**Vasunilashorn SM et al, 2022** [60]	USA	Case-Control Study	CAM	CHI3L1, PF4, MICA, ADCYAP1, RETN, CD300C, CD274, FCGR3B, PAPPA, TNFRSF1A, PLA2G2A, IL-6, TIMP1, THBS1, CD177, CKM, NAAA, ANP32B, KLKB1, BMP1, C4A, CCL27, CTSV, TNFSF9, CCL11, IL-25, AMN, STX1A, CCL16, CHKB, SERPING1, MIA, CCL27, CDH1, PLG, LRIG3
Kaźmierski J et al, 2021 [61]	Poland	Cohort Study	CAM	MCP-1, CRP
Ye C et al, 2020 [62]	China	Cohort Study	CAM-ICU	IL-6, CHI3L1, S100B, Lp-PLA2, MIF, ICAM-1, VCAM-1, BACE1, a-SYN
Ritchie CW et al, 2014 [63]	England	Cross-Sectional Study	CAM	CRP
Plaschke K et al, 2010 [64]	Germany	Cohort Study	CAM-ICU	IL-6, Cortisol
Szwed K et al, 2021 [65]	Poland	Case-Control Study	CAM-ICU	NSP, GFAP
Erikson K et al, 2019 [66]	Finland	Cohort Study	CAM‐ICU	S100B, IL-6
Yuan Y et al, 2020 [67]	China	Case-Control Study	CAM	IL-1b, IL-6, a-SYN
Khan SH et al, 2022 [68]	USA	Randomized Control Trial	CAM‐ICU	CRP, IL-8, IL-10
Dönmezler S et al, 2023 [69]	Turkey	Case-Control Study	DSM-5	eGFR, proBNP
Wiredu K et al, 2023 [70]	USA	Case-Control Study	CAM	CRP, CH3L1, AACT, TIMP1, FGL1, SAA1, SAA2, IBP2, CAH3, CATB, PEPA3, ACTN1
Su LJ et al, 2023 [71]	China	Cohort Study	CAM-ICU	sTNFR-1, sTNFR-2
**Shyam R et al, 2023a** [72]	India	Case-Control Study	CAM-ICU	S100B
Tsui A et al, 2023 [73]	England	Cohort Study	DSM-IV	CRP
Menzenbach J et al, 2021 [74]	Germany	Cohort Study	CAM‐ICU	CCL2, SDC-1
Boogaard MVD et al, 2011 [75]	Netherlands	Cross-Sectional Study	CAM-ICU	IL-8, MCP-1, PCT, Cortisol, TNF-a, MIF, IL-8, IL-1b, IL-1ra, IL-10
Hall RJ et al, 2013 [76]	Scotland	Cohort Study	CAM	S100B
Xu WB et al, 2019 [77]	China	Case-Control Study	CAM	sFGL2
Chai LV et al, 2021 [78]	China	Cohort Study	CAM	IL-6
Khan BA et al, 2013 [79]	USA	Cohort Study	CAM-ICU	S100B
Mao M et al, 2022 [80]	China	Cohort Study	CAM	PGE2, NFL, S100B, GFAP
Khan BA et al, 2020 [81]	USA	Cohort Study	CAM-ICU	IL-6, IL-8, IL-10, TNF-a, CRP, S100B
Neerland et al, 2016 [82]	Norway	Cohort Study	CAM	CRP, sIL-6R
Girard TD et al, 2012 [83]	USA	Cohort Study	CAM-ICU	MMP-9, Protein C, sTNFR1
Maes M et al, 2022 [84]	Thailand	Cohort Study	DRSR-98	GlutaR, AQP4, HSP60
Pfister D et al, 2008 [85]	USA	Cohort Study	CAM-ICU	CRP, S100B, Cortisol
Cerejeira J et al, 2012 [86]	Portugal	Cohort Study	CAM	CRP, IL-6, IL-8, IL-10
**Plaschke K et al, 2023** [87]	Germany	Cohort Study	ICDSC, CAM-ICU	PON1, IgHV3, C1QC, THBS1, FGG
Wu X et al, 2023b [88]	China	Cohort Study	CAM, MDAS	Aβ42, P-tau, T-tau
Kim HJ et al, 2023 [89]	South Korean	Cohort Study	DSM-5	CRP
Rooij SE et al, 2007 [90]	Netherlands	Cohort Study	CAM	IL-6, IL-8
Wang B et al, 2022 [91]	China	Case-Control Study	CAM	APP
McNeil JB et al, 2019 [92]	USA	Cohort Study	CAM-ICU	PAI-1, IL-6
Klimiec Moskal et al, 2021 [93]	Poland	Cohort Study	CAM-ICU	Gal-3BP
Cape E et al, 2014 [94]	Netherlands	Cohort Study	CAM	IL-1b, IL-1ra
Lindblom RPF et al, 2018 [95]	Sweden	Cohort Study	CAM-ICU	TR4, EZH2, CHI3L1, IL-6, SFRP2, PMP2, RTN4R, GFAP, CX3CL1, ICAM-1
Skrede et al, 2015 [96]	Norway	Cohort Study	CAM	MCP-1
Brattinga B et al, 2022 [97]	Netherlands	Cohort Study	DOS scale	IL-6, IL-10, NGAL
Chen BY et al, 2019 [98]	China	Cohort Study	CAM-ICU	IL-6
Shen H et al, 2016 [99]	China	Cohort Study	CAM, DRS-R98	IGF-1
Shyam R et al, 2023b [100]	India	Case-Control Study	CAM-ICU	CRP
Brown et al, 2023 [101]	USA	Cohort Study	CAM, CAM-ICU, DSM-5	NFL
Klimiec-Moskal et al, 2023 [102]	Poland	Cohort Study	bCAM, CAM-ICU	CRP
Imai T et al, 2023 [103]	Japan	Cohort Study	CAM	IL-6
Khan SH et al, 2023 [104]	USA	Case-Control Study	CAM-ICU, RASS	F9, MAN1A1, AGT, CP, C2, ITIH3

### Quality of the included studies

The JBI quality appraisal checklists provided the quality scores for the included studies (S2 File). The majority of the included studies were of medium/moderate quality (n = 55/78, 70.5%) and high quality (n = 23/78, 29.5%), indicating the robustness of the included studies (S2 File). For instance, among the cohort studies (n = 53), 47 studies were assessed as medium quality on the JBI scale when six were of high quality. Based on the quality appraisal checklists, 11 case-control studies were of high quality and seven were of the medium; among the RCT, two studies were of high, and one study was of medium quality. The two cross-sectional and two longitudinal studies were of high quality. In this review, no study was disqualified because of receiving a low-quality rating.

### Delirium-associated important biomolecules

**Fig 3:**
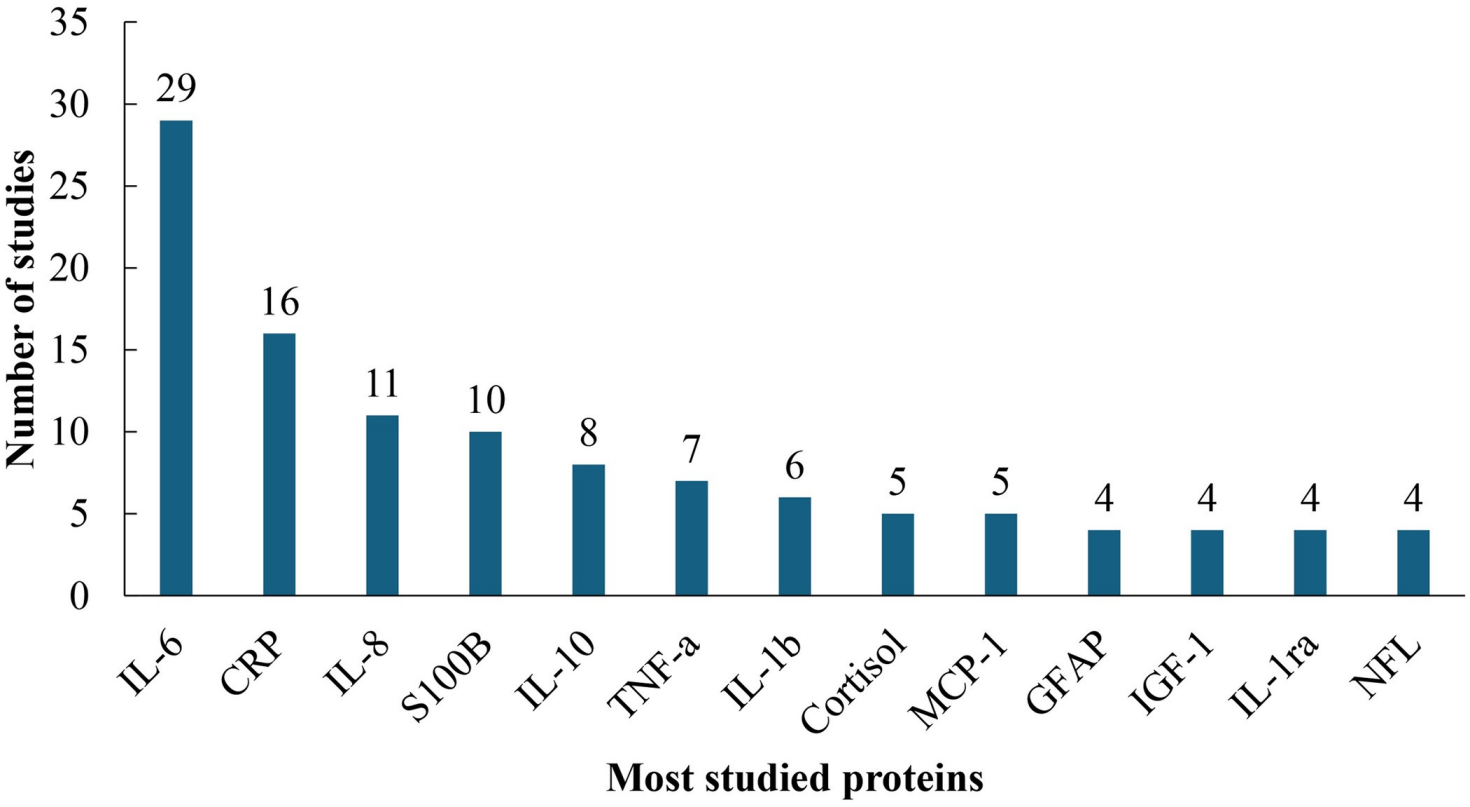
The distribution of the most-studied proteins collected from the included studies.

The included studies showed a total of 313 delirium-associated gene-encoded proteins where there were 189 unique proteins (Table 1 and S4 File). A few proteins were examined repeatedly in a substantial number of studies (Fig 3).

The most studied 13 proteins out of the reported proteins collected from the included studies have been highlighted (Fig 3) in this review as the key common proteins associated with delirium. They are Interlukitin-6 (IL-6), C-reactive protein (CRP), Interlukitin-8 (IL-8), S100B calcium-binding protein, Interlukitin-10 (IL-10), Tumor necrosis factor-a (TNF-a), Interlukitin-1b (IL-1b), Cortisol, Monocyte chemoattractant protein 1 (MCP-1), Glial fibrillary acidic protein (GFAP), Insulin-like-growth-factor-1 (IGF-1), Interlukitin-1 receptor antagonist (IL-1ra), and Neurofilament light polypeptide (NFL). Among them, the IL-6 was mostly reported (n = 29 studies), followed by the CRP (n = 16 studies); IL-8 (n = 11 studies); S100B (n = 10 studies); IL-10 (n = 8 studies); TNF-a (n = 7 studies); IL-1b (n = 6 studies); Cortisol (n = 5 studies); MCP-1 (n = 5 studies); and other proteins subsequently presented in Fig 3.

Based on the distribution of the proteins, we found the top 13 key proteins that were reported in a minimum of four studies. Table 2 describes the basic functionality and clinical justification of the most reported proteins. From the distribution of the functionality of the reported proteins, it has been clear that most of them are associated with cytokines and inflammatory functionality in the human body. Some of them functioned as neurotrophic factors and growth factors. Based on their clinical justification and linkages, they are implicated in a variety of functional pathways, including those for are involved in different functional pathways like the inflammatory response, immune response, neurodegenerative disorder, growth, and brain damage functions, and many more.

**Table 2:** Properties of the 14 most studied proteins.

Annotated Proteins	Full description	Functionality	Clinical justifications
IL-6	Interlukitin-6	Cytokine	Inflammatory marker
CRP	C-reactive protein	Cytokine and Inflammation	Inflammatory marker
IL-8	Interlukitin-8	Cytokine	Inflammatory marker
S100β	S100B, calcium binding protein	Cytokine and Neuronotrophic factor	Tumor marker, brain damage marker
IL-10	Interlukitin-10	Cytokine	Inflammatory marker
TNF-α	Tumor necrosis factor-alpha	Cytokine	Inflammatory marker
IL-1B	Interlukitin-1b	Cytokine	Inflammatory marker
Cortisol	Cortisol	Hormone	Adrenal function indicator
MCP-1	Monocyte chemoattractant protein 1	Cytokine/Chemokine	Inflammation
GFAP	Glial fibrillary acidic protein	Intermediate filaments	Roles in neurodegenerative disorder, cell migration, mitosis, development
IGF-1	Insulin-like-growth-factor-1	Growth factor	Growth hormone deficiency diagnosis, pituitary function indicator
IL-1ra	Interleukin-1 receptor antagonist	Cytokine and Inflammation	Immune and inflammatory responses
NFL	Neurofilament light polypeptide	Filament proteins	Roles in neurodegenerative disorder, interconnection between axons and dendrites

## Discussion

The review showed that most of the significantly associated proteins with delirium belong to the cytokines and inflammatory functionality groups. Although delirium is a multifactorial condition encompassing older age, alcohol or drug usage, severe comorbidities, and anesthesia. A study conducted by Liu et al 2018 [105] showed the cytokines and inflammatory proteins also revealed a strong association of a few of them with delirium.

The included studies reported one or more delirium-associated biomarkers proteins/genes however, there is not enough evidence to justify the use of one specific diagnostic or biomolecule as the sole risk or disease biomarker for delirium. This may happen due to methodological variations, distinct analytical procedures, a variety of patient populations, diagnostic criteria for identifying delirium, and the presence of complicating comorbidities. It was also difficult to determine the strength of the identified relationships between the genes/proteins and delirium.

### Role of cytokines and inflammation in delirium

The included studies revealed a total of 189 unique gene-encoded proteins where some of the proteins occurred frequently such as the proinflammatory cytokines IL-6, IL-8, CRP, S100B, TNF-a, IL-1b, MCP-1, and IL-1ra; the anti-inflammatory cytokine IL-10. The studies suggest that the pro-inflammatory cytokines (PICs), including IL-6 and TNF-a, and anti-inflammatory cytokines, including IL-10, are significant subgroups of inflammatory processes and are crucial in the development of pain sensitivity [51, 106–108]. Since our included studies reported a significant relationship (either positive or negative) between cytokines and delirium development, these indicate that there exists a huge interconnection between cytokines, inflammation, and delirium. The onset of delirium is caused by an early sign of systemic inflammation which indicates the involvement of the cytokine proteins [35, 41, 109–111]. Particularly, regarding its potential to serve as a delirium marker, IL-6 is one of the cytokines that has been examined the most among the included studies. The other cytokines namely, IL-8, MCP-1, IL-1ra, and the anti-inflammatory IL-10 show a significant stimulation of the major immune response pathways as well as in monocyte chemoattraction [29, 94, 96, 112–114]. Plasma IL-6 levels are correlated with delirium intensity and length in critically ill individuals, indicating that systemic inflammation plays a role in the onset and progression of delirium [62, 92]. Our studies suggested that the expression level of cytokine proteins changed significantly in delirious and non-delirious patients or preoperative and post-operative stages. In this aspect, the altered cytokine patterns point to an immune reaction that includes B-cell and T-cell stimulation, immunoglobulin production, and concurrent initiation of anti-inflammatory processes [29, 115, 116].

The CRP plays a novel role in the pathophysiology of delirium as it is associated with stress response, and inflammation and has a role in neurotransmitter activities [82, 117]. Among the investigated studies CRP was reported in 10 individual studies which indicate its significance. Ritchie et al, 2014 reported that CRP has a noticeable association with delirious patients having “musculoskeletal” problems [63]. Although there exists some inconsistency about the role of CRP in delirium as a potential biomarker, it can be focused on future in-depth research to clarify its role broadly.

As a pro-inflammatory cytokine, IL-1b is highly associated with delirium development as well as having a part in the etiology of early delirium [94]. It also plays a vital role in cholinergic activity, a route believed to be responsible for the pathophysiology of delirium [118]. Having some contradictory findings, IL-1b could not be served as a potential individual biomarker for delirium [111]. The S100B is considered as a calcium-binding protein that has involvement in astrocytes with the central nervous system (CNS) and is associated with delirium [66, 119, 120]. The presence of S100B in cerebrospinal fluid (CSF) indicates the early symptoms of Alzheimer’s dementia [75] which is one of the crucial adverse events for delirious patients. A pleiotropic cytokine TNF-a, is associated with several functional pathways including inflammation, necrosis, strong association with cognitive deterioration, apoptosis, and delirium as well [35, 121, 122]. Due to the strong association with cognitive decline (like Alzheimer’s disease), it’s difficult to announce TNF-a as a potential biomarker of delirium [111] which demands further research to clarify the specific role of TNF-a in delirium development.

Other proteins are also found as top studies proteins in our review namely, IGF-1, GFAP, and NFL. All of these are associated with delirium development in preoperative or postoperative stages. IGF-1 is known as a neuroprotective and growth factor which involves in neurogenesis and also may inhibit cytotoxic cytokines, leads pro-inflammation [123, 124]. In our review, three studies reported a negative association of IGF-1 with delirium [28, 91, 124] and one study reported a positive relationship [31]. Due to the linkage with the pathophysiology of Alzheimer’s disease [125, 126], it is still considered an inconsistency among biomarkers of delirium. The study revealed that the likelihood of delirium recovery may be influenced by lower levels of IGF-1 and the lack of the APOE-e4 genotype among female patients [127]. Three studies identify the increased level of GFAP proteins which is significantly associated with delirium [65, 80, 95] in our review. The NFL are associated with ongoing axonal complications and considered as a novel biomarker of Alzheimer’s disease [128, 129], variety of neurological disorders [130, 131].

The above-mentioned discussion indicates the importance of reported significant biomolecules for delirium development. Several cytokines and inflammatory proteins are highly reported mentioning the association with delirium. Future research and deeper molecular investigation should focus on cytokines and inflammation related proteins and their associated signaling pathways to decipher the pathophysiology of delirium.

### Implications

This review concludes that cytokines and inflammatory proteins play a crucial role in delirium development. Delirium’s pathophysiology is multifactorial, and diverse sampling types should be considered for molecular studies [111]. Further studies involving the gene expression study could be a reliable source to identify the differentially expressed genes and proteins associated with delirium. The gene expression data analysis may assist to clarify the pathogenesis of delirium as well as the functional pathways. Future in-depth research on epigenetic analysis and genome-wide association studies may also benefit to identify potential biomarkers which will eventually help in delirium diagnosis and therapeutics. In this regard, this study will be a basis for further proteomic research in delirium.

### Study limitations

This review focused on delirium in humans whether they were identified in ICU or any hospital settings. Studies including Alzheimer’s disease and dementia have not been included to keep the study rigorous and focused solely on delirium. This review’s search for relevant studies may have been limited by the databases used, which might result in the disappearance of potential studies. The current study covered the timeframe between January 2000 and December 2023, therefore, studies before 2000 and after 2023 have not been included. Besides that, this study retrieved published articles from PubMed, Scopus, and EBSCOhost (CINAHL, Medline) databases. If there exist any potential published studies outside of these databases, the article might be missing from this study. The current study focused on accumulating the proteomic biomarkers only, therefore considering the proteins and the gene-encoded proteins to investigate. No specific diagnostic and prognostic proteomic biomarker could be identified through this study. The reported relationship between the proteins and delirium was only considered when the direction of the relationship (positive or negative) was ignored. Therefore, the properties of upregulation or downregulation of proteins could not be described in this review which demands further studies to investigate the differentially expressed genes/proteins identification. Moreover, the cofounding variables, estimation process variation, lack of random allocation, study setting were not considered and completely ruled out.

## Conclusion

This study magnifies the significant information regarding delirium-associated proteomic biomarkers. We have summarized the 13 most studied proteins (IL-6, CRP, IL-8, S100B, IL-10, TNF-a, IL-1b, Cortisol, MCP-1, GFAP, IGF-1, IL-1ra and NFL) about delirium. Notably, cytokine and inflammatory proteomic factors are the most crucial influencer for delirium development and the ultimate stage of delirium, found in this study. Inconsistency among the proteomic biomarkers and the lack of knowledge about the entire pathophysiological process of delirium demand more in-depth molecular studies to decipher the core knowledge of the molecular functionality of delirium. More studies need to be conducted to identify the exclusive causal genomic and proteomic biomarker of delirium which can be investigated as prognostic, diagnostic, and therapeutic target biomolecules. The summarization of the current information on delirium-associated proteins that has been done in this study might serve as a guide for further research and in-depth investigation of delirium.

## Supporting information

S1 File The search sentences are used in different databases and outcomes. (PDF)

S2 File The overall quality appraisal scores. (XLSX)

S3 File The PRISMA checklist. (DOCX)

S4 File Delirium-associated 189 gene-encoded unique proteins. (XLSX)

S1 Table The patient’s demographic and study characteristics table. (DOCX)

S2 Table The screened studies with decision, entire data matrix. (XLSX)
