# Supplementary material for: Accumulating the key proteomic signatures associated with delirium: Evidence from systematic review
Source: PLoS One. 2024 Dec 19;19(12):e0309827. doi: 10.1371/journal.pone.0309827 (PMC11658594; doi:10.1371/journal.pone.0309827)
Supplement: S1 File — (PDF) [file pone.0309827.s001.pdf]

The search sentences used in different databases and number of outcomes from each search

❖ PubMed and MEDLINE Database:

(((((gene[Title/Abstract]) OR (protein[Title/Abstract])) OR (marker[Title/Abstract])) OR (biomarker[Title/Abstract])) AND (delirium[Title/Abstract])) AND (("2000/01/01"[Date - Publication] : "2023/12/31"[Date - Publication]))

Output: Total n=890 articles

❖ Scopus

(TITLE-ABS-KEY (delirium) AND TITLE-ABS-KEY (biomarkers) AND TITLE-ABS-KEY (proteins)) AND PUBYEAR > 1999 AND PUBYEAR < 2024

Output: Total 363 articles

(TITLE-ABS-KEY (delirium) AND TITLE-ABS-KEY (genes) AND TITLE-ABS-KEY (genetics)) AND PUBYEAR > 1999 AND PUBYEAR < 2024

Output: Total 220 articles

❖ EBSCOhost (CINAHL)

(Delirium) AND (genes OR markers OR biomarkers OR proteins OR genetics) AND (publication Date: 20000101-20231231; Exclude MEDLINE records)

Output: Total n=258 articles
