# Supplementary material for: Accumulating the key proteomic signatures associated with delirium: Evidence from systematic review
Source: PLoS One. 2024 Dec 19;19(12):e0309827. doi: 10.1371/journal.pone.0309827 (PMC11658594; doi:10.1371/journal.pone.0309827)
Supplement: S1 Table — (DOCX) [file pone.0309827.s005.docx]

**Supplementary Table 1:** The basic patients’ characteristic information about the selected studies. The age and gender information were considered for delirious patients. If their information was unavailable, then overall sample information was collected.

| **Authors & year** | **Methods of Detecting proteins/Genes** | **Data Collection Time Period** | **Age** | **Sample Size (n)** | **Delirium Patients (n)** | **Gender (n)** |
| --- | --- | --- | --- | --- | --- | --- |
| Adamis et al, 2009 [28] | ELISA protein concentration | July 2003 to April 2004 | M: 84.2(R:70.0-94.0) | 67 | 38 | Fe: 48 [ov] |
| Egberts et al, 2015 [29] | Plasma and Serum Concentration | - | Med: 87.0 (IQR: 84.0, 88.0) | 86 | 23 | Ma: D: 10, ND: 30 |
| Hirsch et al, 2016 [30] | Plasma and CSF Concentration | December 2010 to April 2013 | M: 70.3 (8.7) | 10 | 1 | Fe: D:0, ND: 3 |
| Kazmierski J et al, 2014 [31] | Serum biomarker concentration | May to September 2011 | Med: 68.8 (IQR: 64.0, 74.0) | 113 | 41 | Fe: D; 12, ND: 11 |
| Miao et al, 2018 [32] | Plasma biomarker concentration | July 2012 to June 2015 | M: 71.8(±6.6) | 112 | 49 | Fe: D; 18, ND: 27 |
| Ritter et al, 2014 [33] | Plasma biomarker concentration | February to May 2010 | Med: 56.0 (IQR: 43.0, 75.0) | 78 | 31 | Ma: D: 20, ND:34 |
| Sun et al, 2016 [34] | Plasma biomarker concentration | 2016 | M: 73.2 (±6.5) | 112 | 56 | Fe: D: 29, ND: 33 |
| Van Munster et al, 2008 [35] | Serum biomarker concentration | 2005 to 2007 | M: 84.6 (7.1) | 98 | 50 | Ma: D: 13, ND: 18 |
| Vasunilashorn SM et al, 2015 [36] | Plasma biomarker concentration | - | M: 77.3 (5.0) | 517 | 95 | Fe: D; 54, ND:54 |
| Liu X et al, 2023 [37] | Plasma biomarker concentration | July 2019 to December 2021 | M: 69.2 (±5.0) | 124 | 62 | Ma: D:37, ND: 34 |
| Sun Y et al, 2023 [38] | C-Reactive Protein concentration | July 2021 to July 2022 | M: 70.9 (±6.4) | 643 | 112 | Fe: D: 36, ND: 210 |
| Oren RL et at, 2023 [39] | Plasma biomarker concentration | November 19, 2018 to August 28, 2019 | M: 68.0 (±11) | 76 | 22 | Fe: D: 11, ND: 30 |
| Wu X et al, 2023a [40] | Cerebrospinal Fluid (CSF) concentration | June 2020 to June 2021 | M: 70.2 (±5.2) | 252 | 44 | Fe: D: 44, ND: 190 |
| Ruhnau J et al, 2023 [41] | Serum biomarker concentration | February 2018 to March 2020 | M: 75.7 (±5.8) | 44 | 19 | Fe: D: 11, ND: 12 |
| Westhoff et al, 2013 [42] | Cerebrospinal Fluid (CSF) concentration | March 2008 to March 2009 | M: 84.6 (5.2) | 61 | 23 | Fe: D: 16, ND:26 |
| Heinrich M et al, 2021 [43] | Genome Wide Association Study (GWAS) | October 2014 to April 2017 | Med: 74.0 (IQR:70.0, 76.0) | 745 | 155 | Fe: D: 73, ND: 247 |
| Van Munster et al, 2010 [44] | Single nucleotide polymorphisms (SNPs) | April 2003 to August 2007 | M: 82.3 (7.6) | 720 | 264 | Ma: D: 101, ND: 218 |
| Terrelonge M et al, 2022 [45] | Single nucleotide polymorphisms (SNPs) | 2002 to 2010 | Med: 71.0 (IQR: 67.0, 76.0) | 94 | 47 | Fe: D: 26 |
| Yamanashi T et al, 2021 [46] | DNA Methylation | April 2015 to September 2020 | M: 43.1 (15.1) | 37 | 10 | Fe: D: 5, ND: 8 |
| Yamanashi T et al, 2021 [47] | DNA Methylation | November 2017 to October 2019 | M: 70.5 (10.7) | 77 | 43 | Fe: D:13, ND:14 |
| Steimer M et al, 2021 [48] | Gene expression level comparison | 13 July 2018 to 06 August 2019 | M: 53.54 (16.73) | 48 | 19 | Ma: 42 [ov] |
| Nekrosius D et al, 2019 [49] | Genetic polymorphism analysis | October 2017 to May 2018 | M: 53.0 (17.0) | 48 | 17 | Fe: 10 [ov] |
| Rhee J et al, 2021 [50] | Plasma biomarker concentration | - | M: 75.0 (6.5) | 16 | 8 | Fe: D:5, ND:5 |
| Ballweg T et al, 2021 [51] | Plasma biomarker concentration | 2015 | M: 69.17 (8.10) | 103 | 37 | Fe: D: 18, ND: 27 |
| Tang C et al, 2020 [52] | Plasma biomarker concentration | January 2016 to August 2016 and June 2018 to April 2019 | M: 61.8 (7.5) | 53 | 5 | Ma: 28 [ov] |
| Vasunilashorn SM et al, 2019 [53] | Plasma biomarker concentration | - | M: 77.0 [ov] | 150 | 75 | Fe: 42 [ov] |
| Nübel J et al, 2023 [54] | Neuron-Specific Enolase (NSE) Serum concentration | October 2020 to March 2022 | Med: 79.0 (IQR: 72.0, 84.0) | 141 | 6 | Fe: D:3, ND: 57 |
| Dillon St et al, 2023 [55] | Cerebrospinal Fluid (CSF) concentration | 2009 to 2016 | M: 73.0 (4.9) | 48 | 24 | Fe: D:11, ND:11 |
| Zhang Y et al, 2023 [56] | Plasma biomarker concentration | June 2020 to December 2021 | Med: 81.0 (IQR: 68.0, 85.0) | 126 | 31 | Ma: D:18, ND:38 |
| Liang F et al, 2023 [57] | Plasma biomarker concentration | 2016 to 2020 | M: 73.0 (±5.0) | 139 | 18 | Fe: D:9, ND: 67 |
| Leung JM et al, 2023 [58] | Plasma biomarker concentration | January 2002 to December 2010 | Med: 73.22 (6.06) | 204 | 102 | Fe: D: 65, ND: 66 |
| Van Munster BC et al, 2010 [59] | Plasma biomarker concentration | May 2005 to February 2008 | M: 84.8 (6.9) | 120 | 62 | Ma: D:16, ND:23 |
| Peters van Ton AM et al, 2020 [60] | Cerebrospinal Fluid (CSF) concentration | 2011 to 2016 | M: 64.2 (17.0) | 45 | 15 | Ma: D:5, ND: 8 |
| Vasunilashorn SM et al, 2022 [61] | Plasma biomarker concentration | - | M: 76.4 (±4.3) [ov] | 36 | 18 | Fe: 20 [ov] |
| Ka´zmierski J et al, 2021 [62] | Serum biomarker concentration | April 2017 to November 2019 | Med: 67.0 (IQR: 63.0, 71.0) | 177 | 61 | Fe: D: 15, ND: 24 |
| Ye C et al, 2020 [63] | Plasma biomarker concentration | May 2018 to June 2019 | M: 63.69 (7.21) | 104 | 50 | Ma: D:31, ND: 37 |
| Ritchie CW et al, 2014 [64] | C-reactive protein levels | 4 June 2007 to 4 December 2007 | M: 83.05 (7.4) | 710 | 87 | Ma: D:33, ND:258 |
| Plaschke K et al, 2010 [65] | Plasma biomarker concentration | - | M: 73.3 (±6.0) | 114 | 32 | Ma: 89 [ov] |
| Szwed K et al, 2021 [66] | Plasma biomarker concentration | - | Med: 66.0 (IQR: 63.0, 71.0) | 30 | 12 | Fe: 8 [ov] |
| Erikson K et al, 2019 [67] | Serum biomarker concentration | - | Med: 62.4 (IQR: 49.0, 70.5) | 22 | 10 | Fe: D:6, ND: 2 |
| Yuan Y et al, 2020 [68] | Plasma biomarker concentration | September 2017 to February 2018 | M: 81.0 (6.0) | 202 | 17 | Fe: D: 8, ND: 12 |
| Khan SH et al, 2022 [69] | Serum biomarker concentration | - | Med: 65.9 (IQR: 57.8, 70.6) | 71 | 26 | Fe: D: 7, ND: 7 |
| Dönmezler S et al, 2023 [70] | NA | March, 2021 | M: 69.2 (13.8) | 88 | 45 | Fe: D: 30, ND:15 |
| Wiredu K et al, 2023 [71] | Plasma biomarker concentration | July 2015 to July 2017 | M: 70.0 (±5.0) | 15 | 7 | Ma: D:7, ND:8 |
| Su LJ et al, 2023 [72] | Plasma biomarker concentration | December 2017 to March 2019 | M: 57.0 (10.1) | 318 | 93 | Fe: D: 35, ND: 97 |
| Shyam R et al, 2023a [73] | Serum biomarker concentration | September 2021 to August 2022 | M: 27.5 (±4.9) | 112 | 37 | All Female |
| Tsui A et al, 2023 [74] | Plasma biomarker concentration | January 2017 to December 2018 | M: 81.9 (6.6) | 209 | 115 | Fe: D: 63 |
| Menzenbach J et al, 2021 [75] | Serum biomarker concentration | July to September 2019 | Med: 71.0 (IQR: 66.0, 78.0) | 118 | 33 | Ma: 70 [ov] |
| Boogaard MVD et al, 2011 [76] | Plasma biomarker concentration | February to July 2008 | Med: 72.0 (IQR: 38.0, 86.0) | 100 | 50 | Ma: D: 27, ND: 26 |
| Hall RJ et al, 2013 [77] | Cerebrospinal Fluid (CSF) concentration | 2009 to 2011 | M: 81.3 (6.7) | 45 | 8 | Fe: D: 12, ND: 19 |
| Xu WB et al, 2019 [78] | Serum biomarker concentration | February 2013 to February 2017 | Med: 54.0 (IQR:39.0, 63.0) | 184 | 49 | Fe: D: 16, ND: 41 |
| Chai LV et al, 2021 [79] | Plasma biomarker concentration | March 2018 to January 2020 | M: 56.5 (±11.7) | 221 | 31 | Ma: D: 20, ND: 142 |
| Khan BA et al, 2013 [80] | Serum biomarker concentration | April 2010 to April 2011 | M: 59.0 (±12.6) [ov] | 63 | 63 | Fe: 39 [ov] |
| Mao M et al, 2022 [81] | Serum biomarker concentration | February 1 to June 1, 2019 | Med: 77.0 (IQR: 70.0, 82.0) | 131 | 35 | Fe: D: 20, ND: 57 |
| Khan BA et al, 2020 [82] | Serum biomarker concentration | March 2009–January 2015 | Med: 60.0 (IQR: 52.0, 69.0) | 321 | 321 | Fe: 179 [ov] |
| Neerland et al, 2016 [83] | Cerebrospinal Fluid (CSF) concentration | September 2009 to May 2011 | Med: 85.0 (IQR: 80.0, 89.0) | 149 | 71 | Fe: D: 50, ND: 62 |
| Girard TD et al, 2012 [84] | Plasma biomarker concentration | March 2004 to March 2006 | Med: 66.0 [IQR: 55.0, 75.0] [ov] | 138 | 107 | Fe: 69 [ov] |
| Maes M et al, 2022 [85] | ELISA Assay analysis | June 2019 to February 2020 | M: 85.0 (5.9) | 59 | 23 | Fe: D: 18, ND: 28 |
| Pfister D et al, 2008 [86] | Serum biomarker concentration | January to July 2007 | Med: 74.5 (IQR: 18.0, 90.0) | 16 | 12 | Fe: D: 2, ND: 4 |
| Cerejeira J et al, 2012 [87] | Plasma biomarker concentration | NA | M: 73.0 (±6.3) | 101 | 37 | Ma: D: 15, ND: 35 |
| Plaschke K et al, 2023 [88] | Plasma biomarker concentration | NA | M: 75.1 (±6.9) | 22 | 11 | Fe: D: 3, ND: 3 |
| Wu X et al, 2023b [89] | Cerebrospinal Fluid (CSF) concentration | June 2020 to June 2021 | Med: 74 (IQR: 71.0, 78.0) | 999 | 154 | Ma: D: 91, ND: 495 |
| Kim HJ et al, 2023 [90] | Serum biomarker concentration | January 2014 to December 2018 | M: 80.1 (±7.6) | 300 | 150 | Ma: D: 49, ND:48 |
| Rooij SE et al, 2007 [91] | Serum biomarker concentration | NA | M: 81.2 (7.1) | 185 | 64 | Ma: D: 34, ND: 45 |
| Wang B et al, 2022 [92] | Plasma biomarker concentration | August 2020 to January 2021 | M: 75.2 (±3.5) | 44 | 22 | Fe: D: 10, ND: 11 |
| McNeil JB et al, 2019 [93] | Plasma biomarker concentration | March 2012 to November 2014 | Med: 74.0 (IQR: 68.0, 82.0) | 156 | 64 | Fe: D: 42, ND: 45 |
| Klimiec Moskal et al, 2021 [94] | Plasma biomarker concentration | May 2014 to March 2016 | Med: 79.0 (IQR: 68.0, 84.0) | 160 | 79 | Fe: D: 43, ND: 51 |
| Cape E et al, 2014 [95] | Cerebrospinal Fluid (CSF) concentration | November 2007 to January 2010 and October 2005 to February 2008 | M: 81.3 (6.0) | 43 | 19 | Fe: D: 14, ND: 18 |
| Lindblom RPF et al, 2018 [96] | Serum and Cerebrospinal Fluid (CSF) concentration | NA | M: 59.8 (11.6) [ov] | 23 | 8 | Fe: 5 [ov] |
| Skrede et al, 2015 [97] | ELISA protein concentration | May to December 2006 | Med: 83.0 (IQR: 79.0, 91.0) | 19 | 12 | Fe: 14 [ov] |
| Brattinga B et al, 2022 [98] | Plasma biomarker concentration | 2010 to 2016 | Med: 72.0 (R: 65.0, 89.0) | 311 | 38 | Fe: D: 7, ND: 136 |
| Chen BY et al, 2019 [99] | Serum biomarker concentration | November 2013 to August 2015 | M: 67.0 (7.7) | 266 | 85 | Fe: D: 37, ND: 33 |
| Shen H et al, 2016 [100] | Serum biomarker concentration | March 2013 to May 2015 | M: 73.8 (±5.9) | 140 | 36 | Fe: D:19, ND:61 |
| Shyam R et al, 2023b [101] | Serum biomarker concentration | September 2021 to August 2022 | M: 27.5 (±4.9) | 112 | 37 | All Female |
| Brown et al, 2023 [102] | Plasma biomarker concentration | October 2012 to May 2016 | M: 72.3 (8.1) | 175 | 88 | Ma: D:67, ND: 64 |
| Klimiec-Moskal et al, 2023 [103] | Serum biomarker concentration | May 2014 to March 2016. | Med: 78.0 (IQR: 68.0, 85.0) | 459 | 134 | Fe: D:76, ND: 166 |
| Imai T et al, 2023 [104] | Serum biomarker concentration | September 2016 to February 2021 | M: 72.8 (±7.3) | 221 | 54 | Fe: D: 9, ND: 37 |
| Khan SH et al, 2023 [105] | Serum biomarker concentration | October 2013 to June 2015 | M: 64.6 (9.6) | 52 | 26 | Fe: D:7, ND:6 |

*[Note: M: Mean (±SD: Standard Deviation/R: Range), Med: Median (IQR/R: Range); Ma: Male; Fe: Female; D: Delirium; ND: Non-Delirium; ov: Overall patients]*
